# Foraging behaviour affects nest architecture in a cross-species comparison of ant nests

**DOI:** 10.1098/rstb.2022.0146

**Published:** 2023-08-28

**Authors:** Sean O'Fallon, Kim Drager, Art Zhao, Andrew Suarez, Noa Pinter-Wollman

**Affiliations:** ^1^ Department of Ecology and Evolutionary Biology, University of California, Los Angeles, CA 90065, USA; ^2^ Department of Evolution, Ecology and Behavior, University of Illinois, Urbana, IL 61801, USA; ^3^ Department of Entomology, University of Illinois, Urbana, IL 61801, USA

**Keywords:** nest architecture, foraging behaviour, ant ecology, phylogeny

## Abstract

Animals construct and inhabit nests that can exhibit dramatic intra- and interspecific variation due to differences in behaviour, the biotic and abiotic environment, and evolutionary history. In ants, variation in nest architecture reflects both differences in ecology and in the collective behaviour of the colonies that live in the nests. Each component of the nest (such as depth, and the number, size and connectivity of chambers) reflects selective pressures for different functions, or structural constraints that are imposed by the environment or evolutionary history. To determine potential drivers of nest structure variation in subterranean nests, we performed a meta-analysis of measures of published ant nests to compare different structural elements within and across species. We complemented this survey with 42 nest casts of two closely related species. We quantified nest features that can potentially impact ant foraging behaviour and examined whether phylogeny or foraging strategy are better explanatory variables for the variation we observed. We found that foraging strategy better explained nest features than evolutionary history. Our work reveals the importance of ecology in shaping nest structure and provides an important foundation for future investigations into the selective pressures that have shaped ant nest architecture.

This article is part of the theme issue ‘The evolutionary ecology of nests: a cross-taxon approach’.

## 1. Introduction

Structures built by animals, such as nests, take many forms and fulfill a variety of functions. These functions may include providing shelter, storing food, rearing brood, and attracting mates, and structures can have multiple uses. Nests that some birds build from twigs and feathers are used for rearing offspring, often during one breeding season, and they differ greatly from the elaborate structures that termites excavate and construct, which are used to house entire colonies of thousands of individuals across many years [1]. Building can be energetically costly, yet the abundance of species that construct nests suggests that the benefits gained from this investment outweigh the costs. In addition to being shaped by natural selection, elaborate architectures can act as agents of selection on the behaviour of the organisms that are housed in them [2,3]. Uncovering these selective pressures requires a thorough quantification of the diversity of these structures across species.

While species differ from one another in the types of structures they build, within-species variation also abounds. For example, the environment in which a nest is formed may constrain its architecture due to the availability of building materials or properties of the substrate [4]. In addition, individuals may differ in the way they build because of differences in physiology, behaviour, age, experience, or need. For example, in spotted bowerbirds (*Ptilonorhynchus maculatus*) and bower-building cichlid fish (*Nyassachromis microcephalus*), most males build elaborate structures to attract mates, while ‘subordinate’ or ‘sneaker’ males do not, which may reflect differences in condition, age, or experience [5,6].

Many ant species construct or excavate elaborate nests from different materials and in a variety of environments, resulting in an astonishing diversity of nest form, size, and function [7–9]. Examples of this diversity include using silk from larvae to weave leaves into nests in tree canopies, living in hollowed twigs, branches, or specialized plant cavities, excavating elaborate subterranean nests in soils, and even residing within bivouacs made from the bodies of interlinked workers, as seen in army ants [10]. Nests from species living in the same strata may differ widely in characteristics because of the function the nests provide [11], colony size and demographics [12–15], and the morphology of the workers [16]. For example, species with very similar ecologies can differ in the widths of the tunnels they dig because of variation in worker body size [17], and species with larger colonies excavate nests with more chambers that are better connected than nests of species with smaller colonies [11]. Generally, both the size and number of nest features increase with colony size and over time [14,18]. The environment in which the nest was excavated can also influence nest characteristics [19,20]. For example, nests that penetrate denser soils have chambers that are smaller and closer together than nests in soils that are easier to excavate [21]. Despite the astounding diversity of ant nest structures, little is known about the selective pressures that have resulted in this diversity.

Ant nests serve a variety of functions, and nest features differ in the way they impact these functions. For example, the structure of nest chambers can differ based on their use—with chambers used for growing fungus in *Atta* leaf-cutter ants being oval-shaped, while waste chambers are conical [22]. Tunnels are used for moving within the nest and their length and connectivity may impact the speed of transport [11]. Chambers differ in the degree to which they are connected to one another; many subterranean nests are ‘top heavy’, with many highly connected chambers near the soil surface and ‘chains’ of less connected chambers deeper inside [8,9]. Collective foraging can be influenced by the nest features that are close to the soil surface because recruitment of foragers to food often happens inside the nest—near the nest entrance [23]. Indeed, the connectivity of the entrance chamber (the chamber closest to the nest entrance) expedites recruitment to food [24,25] and the number of nest entrances reduces recruitment speed [26] but allows for a homogeneous exploitation of multiple food sources [27,28]. There are many ways to describe and quantify ant nests, and which features are examined will be determined by the biological question being investigated. Because nest features differ in their function, they might be subject to different selective pressures and therefore some features might be highly conserved while others might differ widely among species and even across nests within a species.

We use ground-dwelling ant species that excavate their nests in soils to examine which nest features differ most across species, and to determine whether these differences can be explained by evolutionary history (i.e. phylogeny) or by natural history (i.e. foraging ecology). Specifically, we ask which nest features differ more among species than within species and which nest features might be conserved, showing as much, or more, variation within a species than across species. We then relate variation in nest features to a species' foraging strategy to determine if foraging strategy or phylogeny better explains variation among species in nest features. Identifying which nest features are influenced by natural history and which ones are constrained by evolutionary history will create a foundation for further research on the selective pressures that shape the evolution of ant nest architecture.

## 2. Methods

### (a) Collection and curation of ant nests from the literature

To compare nest structures across ant species, we augmented a database of previously published nest architecture data and images. We began with the dataset used in Miller *et al*. 2022 [11], which contains chamber counts of 296 nests from 43 species in 24 genera, chamber widths from 188 nets from 37 species in 21 genera, and networks of 170 nests from 38 species in 21 genera. We added newly published nest images to the dataset used in Miller *et al*. 2022 [11] by searching the terms ‘ant nest’, ‘ant nest architecture’ and ‘ant nest excavation’ on Google Scholar. We included in our meta-analysis manuscripts from which we could extract the nest features that are detailed below from three or more nests of the same species. We extracted data from figures, tables and electronic supplementary material. To facilitate testing for intraspecific versus interspecific differences, we excluded species for which we had fewer than three nests quantified. Our final dataset consisted of 397 nests from 31 species in 18 genera (details in the electronic supplementary material, table S1).

### (b) Collection and curation of additional ant nests

To augment data collected from the published literature, we made casts of 42 nests of *Dorymyrmex bureni* (*n* = 10) and *Dorymyrmex elegans* (*n* = 32) near the top of Red Hill at Archbold Biological Reserve near Venus, Florida between July and September of 2018–2019. *Dorymyrmex bureni* and *D. elegans* have workers of similar body size (2–4 mm) and colony size (approx. 1000 individuals) [29,30] and are likely sister species [31]. In addition to providing new taxa for the analyses, these samples provided the opportunity to examine intraspecific variation in nest characteristics, and to explore how nest features differ between closely related taxa. Nest casts were made with zinc using a portable propane-powered field kiln [32]. Excavated nest casts were digitized with a NextEngine 3-D scanner (NextEngine Desktop 3D Scanner Model 2020i, NextEngine, Inc., Santa Monica, CA) using ScanStudio PRO software (v. 2.0.2, NextEngine, Inc., Santa Monica, CA), and broken cast pieces were reconstructed using Geomagic Studio (v. 2014.3.0.1781, 3D Systems, Inc., Rock Hill, SC). After digitization, all nests were included in the previously collected dataset for each nest feature analysed.

### (c) Quantifying nest features

**Figure 1.**
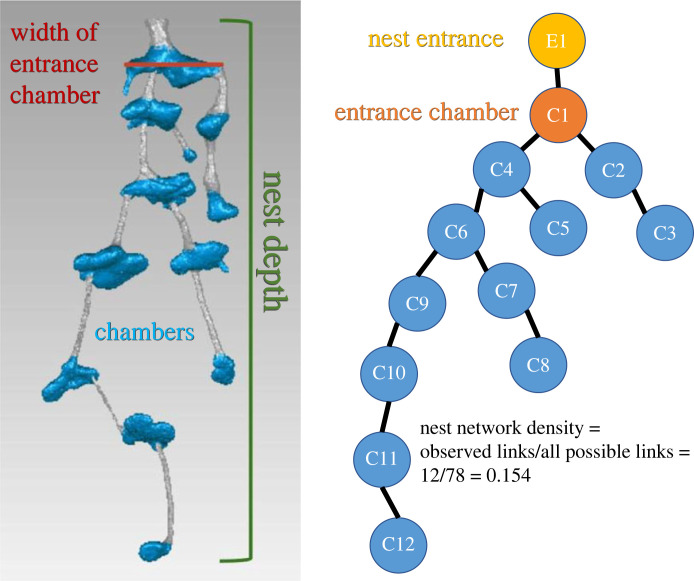
Nest features measured in this study, with a *Dorymyrmex elegans* nest cast as an example. Nest depth (green bracket) is the straight-line distance from the ground surface to the deepest point of the nest. Chambers (blue) are connected by tunnels (grey). Network density is defined as the number of actual connections between nodes divided by the number of possible connections and the network representation of this nest is on the right. Entrance chamber width (red line) is defined as the maximum width of any chamber that is directly connected to the nest entrance.

To characterize nest architecture, we measured features that might impact the way in which ants move inside the nest and that might impact their collective behaviour (figure 1).

#### (i) Depth of the nest

Nest depth is important for the internal thermal environment of the nest, which may affect the colony's ability to thermoregulate [19]. Nest depth was defined as the straight-line distance from the ground surface to the deepest point of the nest. We included nests from papers if authors reported the depth that nests reached below the surface or if authors included scaled photos of nests with the top and bottom of the nest visible. Nest depth was taken directly from published datasets or measured from images using ImageJ [33]. For the *Dorymyrmex* nest casts, we used Geomagic Studio's Analysis tools to measure nest depth. We further scaled nest depth by worker size, to account for morphological differences among ant species. To obtain scaled nest depths, we measured Weber's length (the diagonal length of the mesosoma in profile) from specimens from AntWeb.org [34] with ImageJ. We report results for both nest depth and scaled nest depth.

#### (ii) Total number of chambers

The number of chambers in the nest is important for the nest's capacity to store ants and resources, and suggests the extent to which a colony's worker population can be subdivided into smaller groups. We defined a chamber as a part of the nest where a tunnel widens substantially to produce a disc-like, oval or globular structure, and counted the number of these features to determine the total number of chambers in the nest. We included nests from papers in which the authors reported chamber numbers or photos of nests in which every chamber was visible. For the *Dorymyrmex* nests cast in this study, we counted the number of chambers visible in the digitized cast images.

#### (iii) Nest network density

Network density may be important for the flow of ants and materials throughout the nest. However, if networks are too dense—i.e. there are too many connections—this might have a negative impact on the robustness of the nest, resulting in structural instability and the collapse of tunnels or chambers. We defined nest network density as the number of observed tunnels connecting chambers divided by the number of all possible connecting tunnels. To quantify network density, we first represented nests as networks in which chambers, nest entrances, tunnel junctions and ends of tunnels (if they terminate without reaching a chamber) are nodes, and tunnels are edges that connect those nodes. We then calculated the density of the network using the function edge_density() in the R package igraph [35]. We included nests from papers in which authors reported which chambers were connected by tunnels or provided photos or diagrams with all chambers and tunnels visible. For the *Dorymyrmex* nests cast in this study, we constructed networks based on the digitized cast images of each nest.

#### (iv) Width of entrance chamber

Entrance chamber size is important for the way a colony interfaces with the external environment, and we used its width as an established proxy for chamber volume [11]. We defined an entrance chamber as any chamber that is directly connected to any nest entrance. We included nests from papers in which the authors reported the width of chambers or provided scaled photos with visible entrance chambers. Entrance chamber width was taken directly from published datasets or measured from scaled images with ImageJ. For the *Dorymyrmex* nests cast in this study, we used Geomagic Studio's Analysis tools to measure entrance chamber width. To scale entrance chamber width by worker size, we obtained images of worker specimens for each species from AntWeb.org [34]. We measured the head width and Weber's Length from those specimen photos with ImageJ [33] and used them to generate two scaled variables—entrance chamber scaled by head width and entrance chamber scaled by Weber's length.

### (d) Foraging strategy

To determine the foraging strategy of each species in our meta-analysis, we searched the literature to find published descriptions of foraging behaviour for the species in our database. We performed the literature search in Google Scholar with the search terms: ‘[species] foraging’, ‘[species] foraging strategy’, ‘[genus] foraging’ and ‘[genus] foraging strategy’. We further used publications cited in the electronic supplementary material, table S1 in the review by Lanan 2014 [36]. We assigned a foraging strategy to each species by grouping the definitions from Dornhaus & Powell 2010 [37] into four main foraging types.

#### (i) Solitary foraging

Foragers leave the nest individually and do not appear to interact while searching for or retrieving food items.

#### (ii) Group recruitment

A forager that returns to the nest with food recruits a group of one or several nestmates to the food source.

#### (iii) Stable trails

Foragers leave the nest in trails that persist for at least a day and can sometimes persist for weeks or months.

#### (iv) Mass recruitment

Foragers that return to the nest with food attract a large number of nestmates to the food source by leaving a pheromone trail on their return trip to the nest, or through other means.

Because these foraging strategies differ in their reliance on recruitment of nestmates, we predicted that they would differ in how they relate to the nest features that we measured. Specifically, we hypothesized that species that rely more heavily on recruitment when foraging would have larger entrance chambers and more highly connected nests. Thus, we predicted that entrance chamber width and network density would be smallest in species that rely on solitary foraging, larger in species that employ group recruitment, and highest in species with stable trails and mass recruitment.

### (e) Data analysis

To test whether the nest features we measured differed more within or among species, we performed ANOVA or Kruskal–Wallis tests for each nest feature. We performed four Kruskal–Wallis tests in which depth (unscaled and scaled by Weber's length), number of chambers, or network density were the dependent variables and species ID was the independent variable. We performed two ANOVAs in which either the log-transformed entrance chamber width scaled by head width or the log-transformed entrance chamber width scaled by Weber's length were the dependent variable and species ID was the independent variable.

To estimate the phylogenetic signal of each nest feature, we computed Pagel's lambda (λ) and Blomberg's *K* for each focal feature using the R package phytools [38]. We calculated the mean value of each feature for each species and used a pruned evolutionary tree from Blanchard & Moreau [39] to set branch connections and lengths. Because complete species-level trees were not available for all species in our dataset, we used only one species per genus, choosing the representative species as the one with the most nests measured for the feature being analysed (including for the two *Dorymyrmex* species), and computed phylogenetic signals based on a genus-level tree. To ensure that sub-setting the species in a genus-level analysis did not bias our estimates, we re-ran the computation of the phylogenetic signal with every possible combination of species as representatives of their respective genera and present the range of combinations in the results.

To determine the relationship between foraging strategy and nest structure, we performed ANOVA or Kruskal–Wallis tests for each nest feature. Because we found little to no phylogenetic signal in focal traits, we performed these tests without phylogeny as a factor. We performed four Kruskal–Wallis tests in which depth (unscaled and scaled by Weber's length), number of chambers, or network density were the dependent variables and foraging strategy was the independent variable. We performed two ANOVAs in which either the log-transformed entrance chamber width scaled by head width or the log-transformed entrance chamber width scaled by Weber's length was the dependent variable and foraging strategy was the independent variable. To determine which foraging strategies differed from one another, we performed pairwise *post hoc* comparisons with Tukey's test for features tested with ANOVA and Dunn's test for features tested with Kruskal–Wallis.

All statistical analyses were performed using R (R Core Team, v. 4.0.2 [40]). Data and analysis code are available from the GitHub repository: https://github.com/seanofallon/nest_arch_comp_analysis.

## 3. Results

We found significant differences among species for each nest feature tested in this study. Species differed significantly in total nest depth (Kruskal–Wallis: d.f. = 22, *n*_nest_ = 290, *n*_species_ = 23, *χ*^2^ = 199.403, *p*-value < 0.001; table 1), nest depth scaled by Weber's length (Kruskal–Wallis: d.f. = 22, *N*_nest_ = 290, *N*_species_ = 23, *χ*^2^ = 187.6, *p*-value < 0.001; table 1), number of chambers (Kruskal–Wallis: d.f. = 27, *N*_nest_ = 310, *N*_species_ = 28, *χ*^2^ = 204.9, *p*-value < 0.001; table 1), network density (Kruskal–Wallis: d.f. = 20, *N*_nest_ = 237, *N*_species_ = 21, *χ*^2^ = 174.2, *p*-value < 0.001; table 1) and entrance chamber width scaled by head width (ANOVA: d.f. = 20, *F* = 14.5, *N*_nest_ = 307, *N*_species_ = 21, *p*-value < 0.001; table 2) and by Weber's length (ANOVA: d.f. = 20, *F* = 18.2, *N*_nest_ = 307, *N*_species_ = 21, *p*-value < 0.001; table 2). Comparing *Dorymyrmex* nest casts, the two species differed in number of chambers and nest depth: *Dorymyrmex elegans* nests were deeper (*bureni*: 22.2 cm ± 5.7 s.d., *n* = 9; *elegans*: 38.4 ± 16.9, *n* = 32; d.f. = 39, *t* (two-tailed) = 2.46, *p* = 0.018) and had more chambers (*bureni*: 6.7 ± 1.9 s.d., *n* = 9; *elegans*: 8.6 ± 2.4, *n* = 32; d.f. = 39, *t* (two-tailed) = 2.23, *p* = 0.031).

**Table 1.** Summary statistics of the Kruskal–Wallis tests used to test for interspecific differences in nest depth (unscaled and scaled for Weber's length), number of chambers and nest network density.

feature name	d.f.	Kruskal–Wallis chi-squared	*p*-value
nest depth	22	199.403	<0.0001
scaled nest depth	22	187.600	<0.0001
number of chambers	27	204.913	<0.0001
network density	20	174.159	<0.0001

**Table 2.** Summary statistics of the ANOVAs used to test for interspecific differences in entrance chamber width (ECW) scaled for either head width or Weber's length.

feature name	d.f.	sum Sq	*F*-value	*p*-value
ECW / head width	20	50.901	14.492	<0.0001
ECW / Weber's length	20	64.032	18.231	<0.001

**Figure 2.**
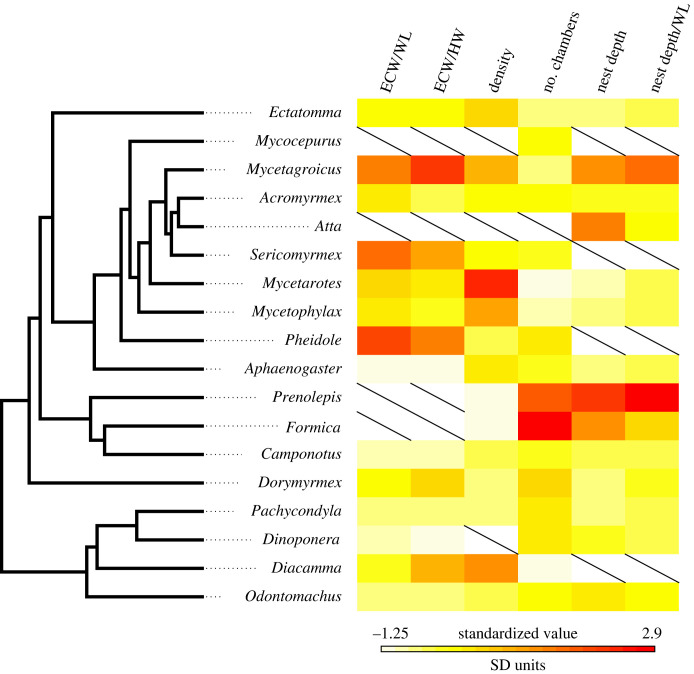
Heatmap showing the mean value for each nest feature in the species representing each genus in the main subset, mapped onto a genus-level phylogeny from Blanchard & Moreau 2017 [39]. Abbreviations indicate: entrance chamber width (ECW), head width (HW), and Weber's length (WL). For a similar plot showing the coefficient of variation for each nest feature, see electronic supplementary material, figure S1.

**Figure 3.**
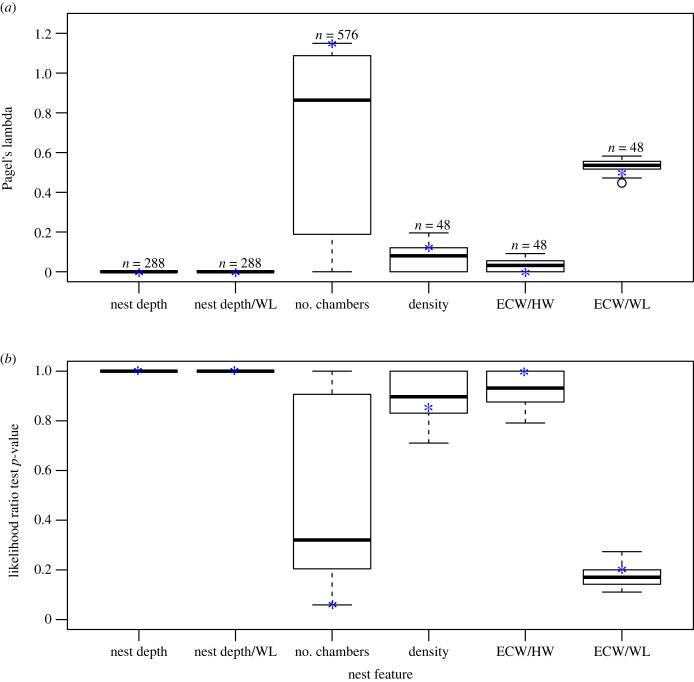
Pagel's lambda (*a*) and the *p*-values from the accompanying Likelihood ratio tests of phylogenetic signal (*b*) from all iterations of phylogenetic analysis that used different subsets of species. The number of subsets used for each feature is indicated above each boxplot in (*a*). Blue asterisks indicate values for the representative phylogenetic tree reported in the main text. Here and in the following figure, horizontal lines are medians, boxes extend to 25 and 75 percentiles, whiskers extend to 1.5 times the interquartile range and dots are outliers. For values of Blomberg's *K* of the phylogenetic analysis, see electronic supplementary material, table S2 and figure S2. Abbreviations indicate: entrance chamber width (ECW), head width (HW), and Weber's length (WL). (Online version in colour.)

We did not detect a phylogenetic signal for any of the measures we examined, except for the number of chambers (figure 2, table 3). For the main subset of species used to compute the phylogenetic signal, we did not detect a phylogenetic signal for nest depth (*λ* < 0.001, likelihood ratio test *p*-value = 1; table 3), nest depth scaled by Weber's length (*λ* < 0.001, likelihood ratio test *p*-value = 1; table 3), network density (*λ* = 0.115, likelihood ratio test *p*-value = 0.851; table 3), entrance chamber width scaled by head width (*λ* < 0.001, likelihood ratio test *p*-value = 1; table 3) and scaled by Weber's length (*λ* = 0.497, likelihood ratio test *p*-value = 0.199; table 3). We found a slight phylogenetic signal for the number of chambers (*λ* = 1.14, likelihood ratio test *p*-value = 0.059; table 3). When examining all possible phylogenetic trees, with all possible species subsets, the *λ* and likelihood ratio test *p*-values were similar to those of the representative species subset (figure 3); for values of Blomberg's *K* of the phylogenetic analysis, see electronic supplementary material, table S2 and figure S2.

**Table 3.** Calculation of phylogenetic signal for each nest feature using the evolutionary tree with a single representative species—see §2e for how these representative species were selected. Pagel's lambda is the signal calculated according to Pagel's lambda and *p*-value corresponds to the *p*-value generated by the likelihood ratio test that accompanies the calculation of *λ*. Abbreviations indicate: entrance chamber width (ECW), head width (HW), and Weber's length (WL).

feature name	Pagel's lambda	*p*-value
nest depth	<0.0001	1.000
nest depth / WL	<0.0001	1.000
no. chambers	1.141	0.059
density	0.115	0.851
ECW / HW	< 0.0001	1.000
ECW / WL	0.497	0.199

**Figure 4.**
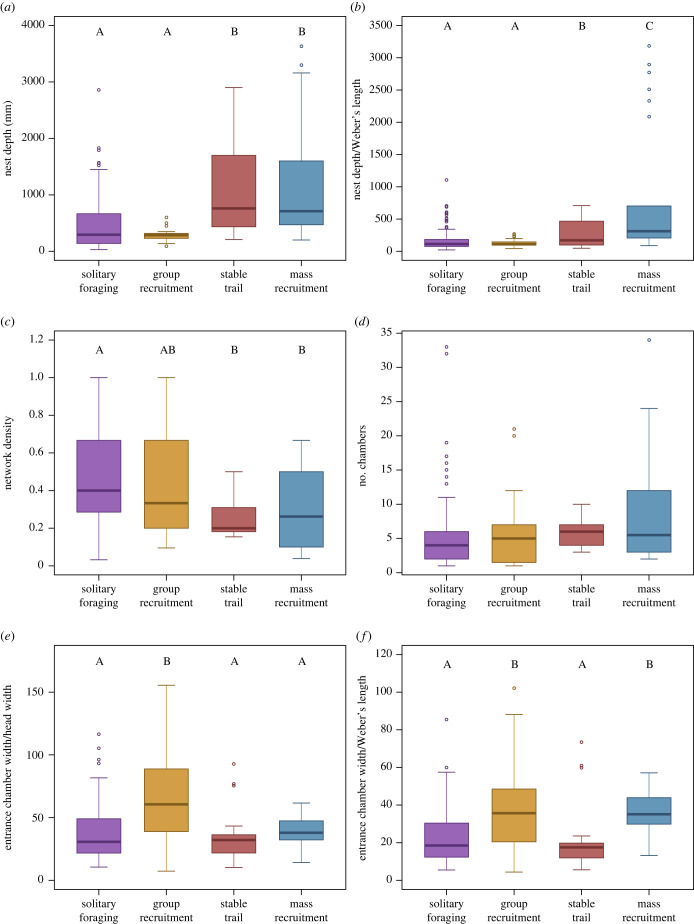
Relationship between nest features and foraging strategy. Each panel shows the relationship between the four foraging strategies that we measured and a different nest feature: (*a*) nest depth in mm; (*b*) nest depth scaled to Weber's length (both measured in mm); (*c*) network density; (*d*) number of chambers; (*e*) width of the entrance chamber scaled to head width (both measured in mm) and (*f*) width of the entrance chamber scaled to Weber's length (both measured in mm). Different letters above the boxes indicate statistically significant differences among foraging strategies according to *post hoc* tests (*p* < 0.05).

All the nest features that we examined were different among foraging strategies (figure 4). Foraging strategies differed significantly in nest depth (Kruskal–Wallis: d.f. = 3, *N*_nest_ = 242, *N*_species_ = 20, *χ*^2^ = 53.7, *p*-value < 0.0001; table 4, figure 4*a*), nest depth scaled by Weber's length (Kruskal–Wallis: d.f. = 3, *N*_nest_ = 242, *N*_species_ = 20, *χ*^2^ = 36.4, *p*-value < 0.0001; table 4, figure 4*b*), network density (Kruskal–Wallis: d.f. = 3, *N*_nest_ = 201, *N*_species_ = 19, *χ*^2^ = 22.6, *p*-value < 0.0001; table 4, figure 4*c*), number of chambers (Kruskal–Wallis: d.f. = 3, *N*_nest_ = 262, *N*_species_ = 25, *χ*^2^ = 10.8, *p*-value = 0.013; table 4, figure 4*d*) and entrance chamber width scaled by both head width (ANOVA: d.f. = 3, *F* = 26.3, *N*_nest_ = 223, *N*_species_ = 17, *p*-value < 0.0001; table 5, figure 4*e*) and Weber's length (ANOVA: d.f. = 3, *F* = 29.4, *N*_nest_ = 237, *N*_species_ = 21, *p*-value < 0.0001; table 5, figure 4*f*).

**Table 4.** Summary statistics of the Kruskal–Wallis tests used to test for differences across foraging strategies in nest depth (unscaled and scaled for Weber's length), number of chambers and nest network density.

feature name	d.f.	Kruskal–Wallis chi-squared	*p*-value
nest depth	3	53.721	<0.0001
scaled nest depth	3	36.433	<0.0001
number of chambers	3	10.764	0.013
network density	3	22.590	<0.0001

**Table 5.** Summary statistics of the ANOVAs used to test for interspecific differences in entrance chamber width (ECW) scaled for either head width or Weber's length.

feature name	d.f.	sum Sq	*F*-value	*p*-value
ECW / head width	3	271.447	26.274	<0.0001
ECW / Weber's length	3	119.076	29.387	<0.0001

## 4. Discussion

Nest features differed among species and some nest features corresponded to the ants' foraging strategy. However, we did not detect a strong phylogenetic signal for the features we analysed. These results suggest that for the soil nesting ants that we examined, variation in nest structure is more likely to reflect differences in natural history, ecology, and the environment than evolutionary history.

Broadly, the nesting biology of ants is phylogenetically conserved as many ant genera, and even subfamilies, have similar nesting habits. For example, Army ants in the subfamily Dorylinae represent a single, conserved transition to nomadism [41], and ants in the subfamily Pseudomyrmicinae live in hollow twigs, branches and other plant cavities with specialized host plant associations (‘myrmecophytes’ like *Acacia* and *Triplaris*) evolving at least a dozen times [42]. Only a few species of *Pseudomyrmex* have reverted to ground-nesting habitats [43]. There are also many examples of genera that are entirely arboreal (e.g. *Cephalotes*, *Oecophylla*) or soil nesting (e.g. *Formica*, and seed harvesting ants in the genera *Pogonomyrmex*, *Messor* and *Veromessor*). However, despite these similarities in nest substrate within phylogenetic clades, there can be great variation among, and even within, species in the characteristics of the nests they make. Our work focused on ant species that nest in the soil, which allows great variation in excavation and construction patterns. It is possible that the physical constraints on excavating soil, such as rocks and plant roots, or soil friability, which may impact the structural stability of the nest, have a greater impact on nest architecture than phylogenetic origin. An investigation into broader aspects of ant nesting biology, like whether nests are arboreal or subterranean, and inclusion of a larger number of species that extend beyond those that nest underground, might reveal a stronger phylogenetic signal than the one we found.

The functional importance of different nest features might shed light on whether they evolve rapidly due to behavioural or environmental variation or are constrained by phylogenetic history. The nest features we examined influence a variety of colony functions. For example, nest depth might relate to thermoregulation, number of chambers may impact a colony's ability to store food and brood, network density can affect movements and interaction frequency, and the size of the entrance chamber can impact the way in which colonies interface with the outside environment, for example, when foraging for food. Species may differ in the importance of these functions, based on their ecology and natural history. Furthermore, nest features may differ in the magnitude of the impact they have on colony function. Thus, different structural elements of the nest might be subject to different selective pressures that depend on the biology of the colony and the way in which each feature impacts colony behaviour. Other nest features might be more, or less, diverse than the ones we measured here, depending on whether they have functional significance and on the functional and evolutionary forces that shape them. Future work might investigate how additional nest features, beyond those that we examined here, differ across species and how they relate to colony function and ecology.

Relying on published work has some limitations. Because the publications we use mostly do not report colony age, we could not account for the effect of colony age on the nest features we examined. Still, when possible to determine, we excluded studies of developing colonies and attempted to only include nests of mature colonies. However, even the nests of mature colonies can change over time and these changes are reflected as variation among nests within the same species in our dataset. Future work on the ontogeny of nest characteristics will provide insight into how developmental and genetic factors interact with environmental variation to shape architecture. Furthermore, given variation in the viscosity of materials used to cast nests, some materials (such as molten aluminium and zinc) go deeper into the nest than other materials (such as plaster) [32]. Thus, it is possible that not all the casts used in this study represent the full structure of subterranean nests and some of the nest depth measurements we used might be underestimates.

Because heat conduction differs in soil and air, deep nests might buffer changes in air temperature. Previous work has investigated the impact of temperature and humidity on nest excavation with mixed results. For example, colonies of the ant *Formica podzolica* dig deeper nests when surface temperature is warm compared to when it is cold [19]. However, the structure and depth of nests dug by the harvester ants *Pogonomyrmex californicus* and *Veromessor andrei* are not affected by temperature or by humidity [17]. In our current work, some of the deepest nests come from species in the genera *Formica* and *Prenolepis* (figure 2), which tend to live in cold environments. Thus, it is possible that species from cold environments tend to dig deeper nests to avoid cold surface temperatures or large seasonal temperature ranges. Further studies that compare nest structures both within and among species across latitude and altitude gradients that correspond to temperature gradients might shed light on the impacts of temperature on nest structure. In addition, future work might examine the effect of temperature on the depth of the shallowest chamber in the nest or on the range of chamber depths.

We found a strong relationship between the nest features we examined and species' foraging strategy. Collective foraging behaviour is a predominant colony function that closely interfaces with nest structure. Past work has shown links between nest structure, specifically the structure and connectivity of the entrance chamber, and the speed at which workers are recruited to forage [24–27]. This link emerges from the impact of physical structures on the movement and interaction rate of ants [44], which underlies the regulation of collective foraging [23,45]. We indeed found that species in which foragers recruit nestmates to food have larger entrance chambers, which potentially allow for more efficient recruitment, compared to species that use solitary or stable trail foraging strategies (figure 4*e,f*). In solitary foraging, there is no recruitment. When there is an established foraging trail, foragers can recruit nestmates to new food directly from the stable trail rather than, or in addition to, from inside the nest [46]. Thus, species that employ solitary or stable trail foraging strategies likely do not require large spaces at the entrance of the nest to recruit nestmates to food. Instead, they can maintain smaller entrance chambers that potentially reduce the risks imposed by invading competitors. Future work on the tradeoffs between foraging and predation, or competition, might shed light on the function of nest structures, especially those at the top of the nest.

Because social interactions are important for recruiting foragers, we expected highly connected nests, which facilitate movement and interactions among ants, to be found in species that recruit to food. However, we found the opposite of this prediction and nests of solitary foragers had the most dense networks, which was the measure we used to quantify nest connectivity (figure 4*c*). While network density may be important for the flow of ants and materials throughout the nest, networks that are too dense, i.e. with too many connections, also have lower structural stability and are prone to the collapse of tunnels and chambers. Indeed, we found a negative, nonlinear, relationship between the number of chambers and nest density (electronic supplementary material, figure S3). Thus, nests with more chambers have relatively fewer connecting tunnels, likely because structural stability becomes more challenging as the number of chambers increases. Some of the most dense networks in our dataset were those of nests with just one or two chambers, which, by definition, have a network density of 1. Solitary foragers tend to have small nests with few chambers (see more below), potentially explaining the opposite relationship we found between foraging strategy and network density. Furthermore, because subterranean nests tend to be top heavy [8,9] with many connected chambers at the top and long ‘chains’ of chambers going deeper, larger nests with deep chamber chains will have lower relative density because the deep chains are not well-connected to the top of the nest. Other measures of nest connectivity might be more suitable than network density to examine the relationship between foraging strategy and nest connectivity. For example, the average shortest path between nest chambers and nest entrance has already been shown to increase with both colony and nest size [11] and therefore might be a suitable measure to relate with foraging strategy.

Measures of nest size were significantly related to foraging strategy. Nests of species that employ mass foraging and stable trail strategies were significantly deeper than those of species that use solitary or group foraging (figure 4*a,b*). Furthermore, foraging strategy significantly related to the number of chambers (figure 4*d*), with numbers of chambers being greater in species with mass and stable trail foraging compared to nests of species that use solitary foraging. Larger nests can house larger colonies (i.e. with more ants) and species with larger colonies have more chambers in their nests [11]. Thus, if species with mass or stable trail foraging tend to have larger colonies than species that use solitary or group foraging [37] then the relationship we found between foraging strategy and nest size could be explained by larger colonies having deeper nests with more chambers. Thus, large nests and mass and stable trail foraging might each be related to colony size for different reasons—to house many ants and to feed many ants—resulting in the correlation between these two colony attributes due to their link to colony size. Future work investigating the relationship between foraging strategy and colony size is needed to examine this hypothesis. It is also possible that species that use mass or stable trail recruitment require more chambers to store the large amount of food they bring back to the nest in bouts of massive foraging events. Number of chambers in the nest had the greatest range of *λ* values when examining different evolutionary trees—with a different species representing each genus in each iteration (figure 3). This large range of *λ* values suggests that there is substantial variation in the number of chambers within genera.

Finally, ant morphology might play a role in the structure of the nest [47]. Previous work found that the composition of body sizes of workers in a polymorphic harvester ant, *Veromessor pergandei*, influences nest structure [16]. In addition, when comparing nest structures excavated by ants from two different harvester ant species, *P. californicus* and *V. andrei,* the species with slightly longer bodies, *V. andrei*, dug wider tunnels, potentially because of the constraints that tunnel width has on the ability of ants to turn around inside the tunnel [17]. Our current work revealed that of the nest features that were scaled to an ant's body size, the strongest phylogenetic signal was found for entrance chamber scaled to Weber's length (figure 3). However, scaling the same nest feature for head width did not reveal a strong phylogenetic signal (figure 3), most likely because head width is not as good a predictor of overall size for some ant subfamilies [48]. As worker size and size variation (worker polymorphism) vary phylogenetically [49], nest features that are most influenced by ant morphology should show the strongest phylogenetic signature. Interestingly, our data suggest that there might be a limit on how small a chamber can be because some of the largest entrance chambers scaled by ant size come from some of the smaller ant species in our dataset (figure 2). Such size limits might emerge from constraints on structural stability that could be impacted by the size of the soil particles and by how tightly they are packed.

Our meta-analysis of ant nests suggests that natural history and ecological function have a stronger impact on nest structure than phylogenetic history. Our findings suggest that nests can be shaped by the ecology in which they have evolved and by selective pressures on the functions that they provide to the animals that built and live in them. Thus, it is important to consider the ecological function of nests when studying their evolutionary history.

## Data Availability

The data and code for analysis are available from the Dryad Digital Repository: https://doi.org/10.5061/dryad.0gb5mkm69 [50] and also from the GitHub digital repository: https://github.com/seanofallon/nest_arch_comp_analysis. Electronic supplementary material can be found on Figshare [51].
